# Comparative Performance of Large Language Models in the Polish State Specialization Examination in Anesthesiology and Intensive Care Medicine

**DOI:** 10.7759/cureus.112868

**Published:** 2026-07-17

**Authors:** Jakub Wojtas, Glieb Pak, Wojciech J Górski

**Affiliations:** 1 Faculty of Medicine, Cardinal Stefan Wyszynski University in Warsaw, Warsaw, POL; 2 Clinical Department of Anaesthesiology and Intensive Care, National Medical Institute of the Ministry of Interior and Administration, Warsaw, POL

**Keywords:** anesthesiology, difficulty index, generative ai, intensive care, large language models, polish language, specialty examination

## Abstract

Background: Large language models (LLMs) are increasingly being explored in medical education and may support clinical knowledge acquisition. However, evidence regarding their performance in non-English, specialty-level national medical examinations remains limited.

Objective: This study aimed to compare the performance of five LLMs in the Polish State Specialization Examination (*Państwowy Egzamin Specjalizacyjny (PES)*) in anesthesiology and intensive care medicine and to assess the association between empirical question difficulty and model accuracy.

Methods: We evaluated ChatGPT 5.2 (OpenAI, San Francisco, CA, USA), Grok 3 (xAI, San Francisco, CA, USA), Claude Sonnet 4.5 (Anthropic, San Francisco, CA, USA), DeepSeek R1 (DeepSeek, Hangzhou, China), and Gemini 2.5 Pro (Google DeepMind, Mountain View, CA, USA) using questions from the autumn 2024 and spring 2025 sessions of the PES in anesthesiology and intensive care medicine. Each session consisted of 120 single-best-answer multiple-choice questions in Polish. The models were prompted to select only one answer option, from A to E. Model accuracy was compared using Cochran’s Q test, followed by post hoc McNemar tests with Holm correction. The association between the difficulty index, derived from examinee performance, and model accuracy was assessed using Spearman’s rank correlation and odds ratios (ORs).

Results: In the autumn 2024 session, model accuracy ranged from 79.2% to 85.8%, with no statistically significant global differences between models. Gemini 2.5 Pro achieved the highest score, answering 103 of 120 questions correctly. In the spring 2025 session, accuracy ranged from 81.7% to 93.3%, and significant global differences were observed between models. Gemini 2.5 Pro achieved the best result, with 112 correct answers out of 120, and significantly outperformed ChatGPT 5.2 and Claude Sonnet 4.5 after the Holm correction. Higher values of the difficulty index were associated with greater model accuracy in both examination sessions. Model performance was higher for questions with difficulty index ≥ 0.5 than for questions with difficulty index < 0.5, 87.4% versus 66.3%, respectively (OR 3.53; 95% CI 2.50-4.99; Wald z = 7.16; p = 7.71 × 10⁻¹³).

Conclusions: Contemporary LLMs achieved high accuracy on Polish specialty-level examination questions in anesthesiology and intensive care medicine. These findings suggest potential educational utility in specialty examination preparation. However, high performance on multiple-choice questions should not be interpreted as evidence of readiness for autonomous clinical use.

## Introduction

Large language models (LLMs) have rapidly become one of the most visible applications of artificial intelligence in medicine. Their potential uses include medical education, information retrieval, summarization of clinical documentation, support for differential diagnosis, and assistance in interpreting complex medical knowledge [1,2,3]. In oncology and other medical fields, LLMs have already been evaluated using examination-style questions, reflecting growing interest in their ability to process and apply domain-specific clinical knowledge [4]. Nevertheless, their use in medicine remains associated with important limitations, including hallucinations, incomplete transparency of training data, inconsistent performance across tasks, and the risk of generating plausible but incorrect answers [5,6,7].

Previous studies have most often evaluated LLMs using English-language medical examinations, particularly general medical licensing or board-style questions. For example, ChatGPT has been tested on United States Medical Licensing Examination (USMLE)-style questions, radiology examinations, ophthalmology assessments, neonatal board-style examinations, and other medical knowledge tests [4,8]. However, the performance of LLMs varies depending on the medical specialty, question type, model version, and language of the prompt. In a large analysis of 2,377 USMLE Step 1-style questions, ChatGPT achieved an overall accuracy of 55.8%, and its performance decreased as question difficulty increased [9]. These findings indicate that examination performance may provide useful information about model capabilities, but it does not fully capture clinical reasoning, safety, or reliability in real-world settings [9,10,11,12].

The language of examination is particularly important. Many LLMs are trained predominantly on English-language data, and several studies have shown that their performance may be lower or less predictable in non-English contexts. German, Polish, Chinese, and Japanese studies suggest that language can influence the accuracy of LLM responses in medical examinations [13,14,15]. In Polish medical examinations, previous studies reported that GPT-4 generally outperformed GPT-3.5 and that English-language prompts often produced better results than Polish-language prompts [13,16,17]. These observations support the need for further evaluation of LLMs in national, non-English medical examination systems. Previous work has also shown that performance on medical MCQs may differ substantially between LLM generations. For example, a study of cardiovascular physiology questions based on standard textbooks reported higher accuracy for ChatGPT-4 than for ChatGPT-3.5, together with more appropriate explanations. These findings support the need for model-specific benchmarking rather than generalizing results from one LLM version to another [18].

Anesthesiology and intensive care medicine represent particularly demanding fields for LLM evaluation. These specialties require integration of physiology, pharmacology, perioperative medicine, mechanical ventilation, hemodynamic monitoring, emergency management, and critical care. Clinical decisions in these areas are often time-sensitive and may have immediate consequences for patient safety. Therefore, evaluating LLMs in anesthesiology and intensive care medicine is relevant not only for medical education but also for understanding the limits of AI-generated medical knowledge in high-risk clinical domains.

The Polish State Specialization Examination (*Państwowy Egzamin Specjalizacyjny (PES)*) is a standardized national examination required for physicians completing specialty training. Although single-best-answer questions do not fully represent real clinical practice, they provide an objective and reproducible benchmark for comparing model performance. The PES has already been used in studies assessing LLM performance in other specialties, including radiology, dermatology, obstetrics and gynecology, ophthalmology, infectious diseases, and vascular surgery [13,16,19]. However, data on the performance of current LLMs in anesthesiology and intensive care medicine remain limited.

The aim of this study was to compare the performance of five contemporary LLMs, such as ChatGPT 5.2 (OpenAI), Grok 3 (xAI), Claude Sonnet 4.5 (Anthropic), DeepSeek R1 (DeepSeek), and Gemini 2.5 Pro (Google DeepMind), on the PES in anesthesiology and intensive care medicine. We also assessed whether model accuracy was associated with empirical question difficulty, as measured by the difficulty index derived from examinee performance.

## Materials and methods

Study design

This study was designed as a comparative cross-sectional benchmark evaluation of LLMs on Polish specialty-level medical examination questions. The analysis assessed the accuracy of five contemporary LLMs in answering single-best-answer multiple-choice questions from the PES in anesthesiology and intensive care medicine.

This examination-based design was selected because multiple-choice questions allow objective scoring and direct comparison between models. Similar approaches have been widely used in previous studies evaluating LLMs in medical and standardized examinations, primarily because closed-format questions reduce subjective assessment bias and enable reproducible comparisons [20,21]. However, this format should be interpreted as an assessment of examination performance rather than as a direct measure of clinical decision-making ability.

Examination data

The dataset consisted of two complete PES sessions in anesthesiology and intensive care medicine: the autumn 2024 session and the spring 2025 session. Each session included 120 Polish-language, single-best-answer multiple-choice questions with five answer options, labeled A to E. In total, 240 examination questions were included.

The official answer key was used as the reference standard for determining correctness. For each question, the difficulty index, derived from human examinee performance, was collected when available. In this study, greater difficulty index values indicated a higher proportion of correct answers among human examinees and therefore lower empirical question difficulty. Accordingly, the difficulty index was interpreted as an inverse measure of difficulty, or as an empirical ease index.

No patient-level data were used. The study was based exclusively on publicly available or institutionally accessible examination materials and aggregated examination statistics. Therefore, formal ethics committee approval was not required.

Evaluated models

Five LLMs were evaluated: ChatGPT 5.2, Grok 3, Claude Sonnet 4.5, DeepSeek R1, and Gemini 2.5 Pro. All models were tested between December 2025 and February 2026. The models were accessed through the Open WebUI platform (Open WebUI, San Francisco, CA, USA), which was connected directly to the models' official provider application programming interfaces (APIs) via API keys to ensure direct model interaction without third-party system prompt modifications or context truncation. Default system prompts of the Open WebUI were disabled, and the default inference parameters were used, with the temperature set to near-zero (e.g., 0.0) to ensure deterministic outputs and minimize run-to-run variability.

Because LLMs are frequently updated, the results should be interpreted as reflecting the performance of the tested model versions at the time of evaluation. This is relevant because previous studies have emphasized that LLM performance may not be fully reproducible after model updates or changes in deployment settings [22].

Prompting procedure

Each question was submitted to each model in Polish in a separate conversation, together with all five answer options. The models were instructed to select the best answer only. No additional clinical context, explanations, hints, or feedback were provided. The models were not asked to justify their answers because the primary outcome was binary correctness according to the official answer key.

Outcome measures

The primary outcome was model accuracy, defined as the proportion of questions answered correctly according to the official answer key. Accuracy was calculated separately for each model and each examination session.

Secondary outcomes included: comparison of accuracy between models within each examination session; pairwise post hoc comparisons between models when the global test was statistically significant; association between difficulty index and model accuracy; and comparison of model performance between questions with difficulty index < 0.5 and difficulty index ≥ 0.5.

The threshold of difficulty index = 0.5 was used to distinguish questions answered correctly by fewer than half of human examinees from questions answered correctly by at least half of human examinees. This dichotomization was selected for interpretability, although it may reduce the information contained in the difficulty index as a continuous variable.

Statistical analysis

Categorical variables were reported as counts and percentages. Model accuracy was reported as the number and percentage of correct answers. Where appropriate, 95% confidence intervals for accuracy were calculated using the Wilson score method.

Because the same set of questions was answered by all models within each examination session, model responses were treated as paired binary data. Global differences in accuracy between models were assessed using Cochran’s Q test. The Q statistic, degrees of freedom, p-value, and Kendall’s W as an effect size measure were reported. When the global test was statistically significant, pairwise comparisons were performed using exact McNemar tests with Holm correction for multiple testing. For exact McNemar tests, discordant pair counts and the exact test statistic, defined as the smaller number of discordant pairs, were reported.

The association between DI and model performance was assessed at the item level using Spearman’s rank correlation coefficient. Per-item model facility (pLLM) was defined as the fraction of models answering a given question correctly. For Spearman correlations, asymptotic t statistics with n − 2 degrees of freedom were reported. In addition, model accuracy was compared between questions with a difficulty index < 0.5 and questions with a difficulty index ≥ 0.5 at the response level. The strength of this association was expressed as an odds ratio (OR) with a 95% confidence interval (CI), and the Wald z statistic for the log OR was reported.

All statistical tests were two-sided, and p-values below 0.05 were considered statistically significant.

Statistical analyses were performed using Python (Python Software Foundation, Wilmington, DE, USA) in a Jupyter Notebook environment (Project Jupyter, NumFOCUS, Austin, TX, USA), with pandas, scipy, statsmodels, matplotlib, and seaborn libraries.

## Results

Dataset characteristics

The final dataset included 240 single-best-answer multiple-choice questions from two PES sessions in anesthesiology and intensive care medicine. These included 120 questions from the autumn 2024 session and 120 questions from the spring 2025 session. Each of the five LLMs answered all questions, resulting in 1,200 model responses in total.

Across both sessions, the models provided 1,006 correct answers out of 1,200 responses, corresponding to an overall accuracy of 83.8%. Accuracy varied by model and examination session. Detailed model-level accuracy estimates for both examination sessions are presented in Table 1. 

**Table 1:** Accuracy of large language models on the autumn 2024 and spring 2025 PES sessions. Accuracy is presented as the percentage of correct answers according to the official answer key. Confidence intervals were calculated using the Wilson score method. Global between-model differences were assessed using Cochran’s Q test. In the autumn 2024 session, Cochran’s Q test showed no statistically significant global difference between models, Q(4) = 3.919, p = 0.4171, Kendall’s W = 0.008. In the spring 2025 session, Cochran’s Q test showed a statistically significant global difference between models, Q(4) = 14.444, p = 0.006004, Kendall’s W = 0.030. CI = confidence interval; PES = Polish State Specialization Examination.

Session	Model	Correct answers, n	Total questions, n	Accuracy, %	95% CI lower	95% CI upper
Autumn 2024	ChatGPT 5.2	99	120	82.5	74.7	88.3
Autumn 2024	Claude Sonnet 4.5	96	120	80.0	72.0	86.2
Autumn 2024	DeepSeek R1	98	120	81.7	73.8	87.6
Autumn 2024	Gemini 2.5 Pro	103	120	85.8	78.5	91.0
Autumn 2024	Grok 3	95	120	79.2	71.1	85.5
Spring 2025	ChatGPT 5.2	98	120	81.7	73.8	87.6
Spring 2025	Claude Sonnet 4.5	99	120	82.5	74.7	88.3
Spring 2025	DeepSeek R1	101	120	84.2	76.6	89.6
Spring 2025	Gemini 2.5 Pro	112	120	93.3	87.4	96.6
Spring 2025	Grok 3	105	120	87.5	80.4	92.3

Model performance in the autumn 2024 examination session

In the autumn 2024 session, model accuracy ranged from 79.2% to 85.8%. Gemini 2.5 Pro achieved the highest score, answering 103 of 120 questions correctly, followed by ChatGPT 5.2 with 99 correct answers, DeepSeek R1 with 98 correct answers, Claude Sonnet 4.5 with 96 correct answers, and Grok 3 with 95 correct answers. Global between-model differences were not statistically significant in this session, Cochran’s Q test, Q(4) = 3.919, p = 0.4171, Kendall’s W = 0.008.

Model performance in the spring 2025 examination session

In the spring 2025 session, model accuracy ranged from 81.7% to 93.3%. Gemini 2.5 Pro again achieved the highest score, answering 112 of 120 questions correctly. Grok 3 answered 105 questions correctly, DeepSeek R1 101 questions, Claude Sonnet 4.5 99 questions, and ChatGPT 5.2 98 questions. Global between-model differences were statistically significant in this session, Cochran’s Q test, Q(4) = 14.444, p = 0.006004, Kendall’s W = 0.030.

Because the global Cochran’s Q test was statistically significant in the spring 2025 session, post hoc pairwise comparisons were performed using exact McNemar tests with Holm correction. The statistically significant pairwise comparisons are presented in Table 2. Gemini 2.5 Pro significantly outperformed ChatGPT 5.2, exact McNemar test statistic = 0, raw p = 0.000122, Holm-adjusted p = 0.00122, and Claude Sonnet 4.5, exact McNemar test statistic = 1, raw p = 0.000977, Holm-adjusted p = 0.00879. All remaining pairwise comparisons were not statistically significant after Holm correction.

**Table 2:** Significant post hoc pairwise comparisons after Holm correction in the spring 2025 session. Pairwise comparisons were performed using exact McNemar tests with Holm correction only for the spring 2025 session, because the global Cochran’s Q test was statistically significant. Model A refers to Gemini 2.5 Pro in both comparisons. For exact McNemar tests, the test statistic is reported as the smaller number of discordant pairs. All remaining pairwise comparisons were not statistically significant after Holm correction.

Comparison	Test	Both correct, n	Model A correct/model B incorrect, n	Model A incorrect/model B correct, n	Both incorrect, n	Exact test statistic	Raw p-value	Holm-adjusted p-value	Interpretation
Gemini 2.5 Pro vs ChatGPT 5.2	Exact McNemar test	98	14	0	8	0	0.000122	0.00122	Significant
Gemini 2.5 Pro vs Claude Sonnet 4.5	Exact McNemar test	98	14	1	7	1	0.000977	0.00879	Significant

Overall performance across both examination sessions

**Figure 1:**
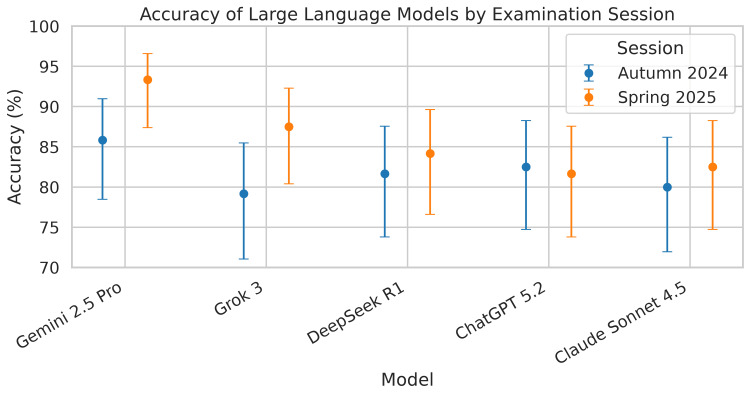
Accuracy of five large language models on the autumn 2024 and spring 2025 PES sessions in anesthesiology and intensive care medicine. Error bars represent 95% confidence intervals calculated using the Wilson score method. PES = Polish State Specialization Examination

Across both examination sessions, Gemini 2.5 Pro achieved the highest combined accuracy, with 215 correct answers out of 240 questions, corresponding to 89.6%. Grok 3 answered 200 of 240 questions correctly, DeepSeek R1 199 of 240, ChatGPT 5.2 197 of 240, and Claude Sonnet 4.5 195 of 240. This combined analysis should be interpreted descriptively because the two examination sessions differed in question content and empirical difficulty distribution. Model accuracy with 95% CIs is shown in Figure 1.

Association between question difficulty index and model accuracy

Model performance was associated with the human-derived difficulty index. At the item level, higher difficulty index values were associated with higher per-item model facility in both examination sessions. In the autumn 2024 session, the Spearman correlation was ρ = 0.362, t(118) = 4.22, p = 4.79 × 10⁻⁵. In the spring 2025 session, the corresponding correlation was ρ = 0.350, t(118) = 4.05, p = 9.02 × 10⁻⁵. Detailed item-level and response-level analyses are presented in Table 3.

**Table 3:** Association between DI and model performance. Higher DI values indicate questions answered correctly by a larger proportion of human examinees and, therefore, lower empirical question difficulty. pLLM was defined as the fraction of models answering a given question correctly. Accuracy by DI group was calculated at the response level, with each model response treated as one observation. CIs for accuracy were calculated using the Wilson score method. Spearman test statistics were calculated using the asymptotic t approximation. The odds ratio p-value was calculated using the Wald test for the log odds ratio. DI = difficulty index; pLLM = per-item model facility; CI = confidence interval; NA = not applicable.

Analysis	Session or comparison	Unit of analysis	Statistical test	n	df	Test statistic	Effect measure	Estimate	95% CI lower	95% CI upper	p-value
DI-pLLM association	Autumn 2024	Question	Spearman rank correlation	120	118	4.22	Spearman ρ	0.362	NA	NA	4.79 × 10⁻⁵
DI-pLLM association	Spring 2025	Question	Spearman rank correlation	120	118	4.05	Spearman ρ	0.350	NA	NA	9.02 × 10⁻⁵
Model accuracy by DI group	DI < 0.5	Model response	Wilson score CI	205	NA	NA	Accuracy (%)	66.3	59.6	72.5	NA
Model accuracy by DI group	DI ≥ 0.5	Model response	Wilson score CI	995	NA	NA	Accuracy (%)	87.4	85.2	89.4	NA
Correct response odds	DI ≥ 0.5 vs DI < 0.5	Model response	Wald test for log odds ratio	1200	NA	7.16	Odds ratio	3.53	2.50	4.99	7.71 × 10⁻¹³

When questions were dichotomized by difficulty index, model accuracy was substantially lower for questions with difficulty index < 0.5 than for questions with difficulty index ≥ 0.5. At the response level, models answered 136 of 205 responses correctly for questions with difficulty index < 0.5, corresponding to 66.3%, compared with 870 of 995 responses for questions with difficulty index ≥ 0.5, corresponding to 87.4%. The odds of a correct model response were higher for questions with difficulty index ≥ 0.5, OR = 3.53, 95% CI 2.50-4.99, Wald z = 7.16, p = 7.71 × 10⁻¹³.

These findings indicate that questions answered correctly by a larger proportion of human examinees were also more likely to be answered correctly by LLMs.

## Discussion

In this comparative study, five contemporary LLMs achieved high accuracy on PES questions in anesthesiology and intensive care medicine. In the autumn 2024 session, all models scored between 79.2% and 85.8%, and no statistically significant global differences were observed. In the spring 2025 session, the accuracy ranged from 81.7% to 93.3%, with significant differences between models. Gemini 2.5 Pro achieved the highest score in both sessions and significantly outperformed ChatGPT 5.2 and Claude Sonnet 4.5 in the spring 2025 session after correction for multiple comparisons.

These results suggest that current LLMs can answer a high proportion of specialty-level medical examination questions in Polish. The observed accuracy rates are higher than those reported in earlier studies of older models, such as ChatGPT-3.5, which often achieved results close to or below passing thresholds in medical examinations. For example, an in-depth analysis of ChatGPT performance on USMLE Step 1-style questions reported an overall accuracy of 55.8% [9]. Polish studies have also shown substantial improvements between ChatGPT-3.5 and GPT-4 in specialty examinations, including obstetrics and gynecology and dermatology [13,16]. The high scores observed in the present study are consistent with the broader trend of rapid performance improvement among newer LLMs.

The findings are particularly relevant because the examination was conducted in Polish. Most LLMs have been trained primarily on English-language corpora, and previous research suggests that model performance may vary across languages [23]. Polish studies comparing responses in Polish and English have generally shown better performance when questions are provided in English, although the magnitude and statistical significance of this difference vary across studies and specialties [13,16,17]. In the present study, all questions were administered in Polish, which strengthens the relevance of the results for the national medical education context. However, it also means that the findings should not be directly extrapolated to English-language versions of the same questions without further testing.

An important observation was the relationship between empirical question difficulty and model accuracy. Higher difficulty index values, indicating lower empirical question difficulty, were associated with better model performance in both examination sessions. Accuracy was substantially higher for questions with a difficulty index ≥ 0.5 than for those with a difficulty index < 0.5, 87.4% versus 66.3%, respectively. This suggests that questions answered correctly by a larger proportion of human examinees were also more likely to be answered correctly by LLMs. Similar associations between question difficulty and model performance have been reported in previous medical examination studies [9]. This finding may indicate that LLMs are more reliable when questions assess well-established, widely represented knowledge, but less reliable when questions require nuanced interpretation, advanced clinical reasoning, or recognition of rare or controversial issues.

The superiority of Gemini 2.5 Pro in the spring 2025 session should be interpreted cautiously. Although the differences were statistically significant for selected pairwise comparisons, the study was based on two examination sessions and a finite number of questions. The pairwise comparisons with ChatGPT 5.2 and Claude Sonnet 4.5 were driven by extremely small counts of discordant pairs (specifically, 0 and 1 discordant pairs, as shown in Table 2). Such low cell counts reduce the statistical robustness of these comparisons, and the apparent superiority might not hold over larger test sets or different question distributions. Differences between models may reflect not only their underlying capabilities but also their training data, update cycles, instruction-following behavior, handling of Polish medical terminology, and sensitivity to prompt format. Furthermore, LLMs are frequently updated, and performance measured at one point in time may not be fully reproducible in the future. This limitation has also been emphasized in other studies evaluating rapidly evolving models [23].

The educational implications of these findings are important. LLMs may be useful as supplementary tools for physicians preparing for specialty examinations. They may assist with self-testing, explanation of concepts, generation of learning summaries, and identification of topics requiring further review. However, the use of LLMs in education should be accompanied by a critical appraisal of generated answers. Previous studies have emphasized that students and physicians should not rely blindly on LLM-generated responses, but should verify them against guidelines, textbooks, and expert opinion [23]. This is especially important in anesthesiology and intensive care medicine, where incorrect recommendations may have serious implications for patient safety.

Despite high examination performance, these results do not demonstrate that LLMs are ready for autonomous clinical decision-making. Multiple-choice questions provide a structured environment with predefined answer options and one correct answer. Real clinical practice is substantially more complex and involves incomplete data, dynamic patient status, uncertainty, competing priorities, ethical considerations, and responsibility for patient outcomes. Therefore, performance on examination questions should be interpreted as evidence of medical knowledge retrieval and pattern recognition rather than proof of safe clinical reasoning.

Future studies should evaluate LLMs using more clinically realistic tasks, including open-ended case vignettes, dynamic scenarios, simulated emergencies, and questions requiring justification of management decisions. It would also be valuable to assess the correctness and safety of model explanations, not only the final answer. In addition, future research should compare model responses with those of physicians at different levels of training and evaluate whether LLM assistance improves or worsens human decision-making.

Limitations

This study has several limitations. First, the analysis was limited to two sessions of a single Polish specialty examination, which may restrict generalizability to other specialties, countries, and examination formats. Second, only single-best-answer multiple-choice questions were evaluated. This format enables objective scoring but does not fully assess clinical reasoning, uncertainty management, prioritization, or patient safety. Third, the models were instructed to provide only the selected answer option; therefore, the quality of reasoning, correctness of explanations, and presence of hallucinations were not evaluated. Fourth, potential data contamination cannot be excluded because some examination questions or similar educational materials may have been publicly available before model testing. Fifth, LLMs are frequently updated, and the results apply only to the model versions available at the time of evaluation. Finally, analyses based on model responses may not fully account for clustering by question and model; therefore, associations between difficulty index and model correctness should be interpreted as exploratory.

## Conclusions

In this comparative benchmark study, five contemporary LLMs achieved high accuracy on PES questions in anesthesiology and intensive care medicine. Model performance was associated with empirical question difficulty, with higher accuracy observed for questions that were easier for human examinees. These findings suggest that LLMs may be useful as supplementary tools for specialty medical education and examination preparation. However, performance on multiple-choice questions should not be interpreted as evidence of readiness for autonomous clinical decision-making. Further studies should evaluate reasoning quality, reproducibility, explanation safety, and performance in realistic clinical scenarios.
